# Development of a Customizable Web-Based Dashboard for Remote Blood Pressure Monitoring: Feasibility and Usability Implementation Study

**DOI:** 10.2196/62700

**Published:** 2025-08-06

**Authors:** Joshua Wollen, Thuy Nguyen, Charles Coton, Gina DeWildt, Gabretta Cooksey, Audrey Cohen, Nicholas Hoang, Munachi Okpala, Michael Gonzalez, Mengxi Wang, Chigozirim Izeogu, Elmer Bernstam, Charles Green, Sean Savitz, Jose-Miguel Yamal, Anjail Sharrief

**Affiliations:** 1 Department of Pharmacy Practice and Translational Research, College of Pharmacy, University of Houston, Houston, TX, United States; 2 Department of Pharmacy, Texas Medical Center, Memorial Hermann–Texas Medical Center, Houston, TX, United States; 3 Coordinating Center for Clinical Trials, School of Public Health, The University of Texas Health Science Center at Houston, Houston, TX, United States; 4 Department of Neurology, McGovern Medical School, The University of Texas Health Science Center at Houston, 6431 Fannin St, MSB 7.110, Houston, TX, 77030, United States, 1 7135006538; 5 Department of Clinical and Health Informatics, McWilliams School of Biomedical Informatics, The University of Texas Health Science Center at Houston, Houston, TX, United States; 6 Health Services Research, College of Medicine, Baylor University, Houston, TX, United States; 7 Department of Pediatrics, McGovern Medical School, The University of Texas Health Science Center at Houston, Houston, TX, United States; 8 Institute for Stroke and Cerebrovascular Disease, UT Health Houston, The University of Texas Health Science Center at Houston, Houston, TX, United States; 9 Department of Biostatistics and Data Science, School of Public Health, The University of Texas Health Science Center at Houston, Houston, TX, United States

**Keywords:** electronic data capture, remote monitoring, telehealth, customization, web based, blood pressure, stroke, heart, development, video based, patient management

## Abstract

**Background:**

Effective blood pressure (BP) management is essential for several disease states, including secondary stroke prevention. Remote BP monitoring used in combination with an electronic data capture (EDC) system offers potential for improved BP control for patients. While such systems currently exist, there are many patient-, provider-, and system-specific challenges these systems currently do not address. In addition, such systems are rarely described transparently and comprehensively in the literature with enough detail to be reproduced by other clinicians.

**Objective:**

This study aims to describe the development and design of a web-based EDC system tailored for remote BP monitoring and management to overcome common patient-, provider-, and system-specific challenges.

**Methods:**

The EDC system was developed for use in the Video-based Intervention to Reduce Treatment and Outcome Disparities in Adults living with Stroke (VIRTUAL) and transient ischemic attack clinical trial, a randomized study involving adult stroke survivors receiving care for secondary stroke prevention. The EDC system supports remote monitoring using a BP monitor, which was specifically and methodically chosen for its suitability to the population. The EDC system integrates the data from the remote monitors into a web-based application, facilitating real-time monitoring for health care team intervention. The system includes a BP dashboard displaying time-series data, visual alerts, and email notifications for out-of-range readings. Proxy measures assessed system effectiveness in addressing patient-, provider-, and system-specific needs.

**Results:**

Seventeen BP monitors were evaluated, and the BlipcareBlip BP800 was selected based on its cellular connectivity, ergonomic design, and application programming interface compatibility. The EDC system successfully linked patient data to the dashboard, enabling real-time visualization of BP trends and provider alerts. A total of 97% (184/190) of participants in the intervention arm engaged in BP monitoring, averaging 1.2 readings per day. Clinicians actively used the system, with mean monthly logins ranging from 19 to 44 per provider. Alerts were triggered for 69% (131/190) of participants, primarily for systolic BP abnormalities. Data transmission delays were minimal, with a median time of 1.4 minutes from data collection to dashboard entry. Outlier alerts were efficiently processed, with an average notification time of 17.9 hours. The system demonstrated high functionality in addressing patient-, provider-, and system-specific needs, supporting effective BP monitoring and intervention delivery.

**Conclusions:**

The VIRTUAL EDC system facilitates remote monitoring of BP in stroke survivors, offering a model for similar systems in clinical trials. Clinicians can analyze, track, and interpret monitored data in near real-time, remotely, which may enhance BP management approaches, particularly in communities with limited access to care. This approach has the potential to improve hypertension management for patients and clinicians when standard data transparency is insufficient, standard monitor features are incompatible with the user, or when integrating a commercial dashboard may threaten electronic health record security.

## Introduction

Elevated systolic and diastolic blood pressure (BP) are associated with an increased risk for stroke [1]. An American Heart Association (AHA) Presidential Advisory Committee projected that hypertension prevalence will increase from 51.2% in 2020 to 61.0% in 2050, leading to higher rates of cardiovascular disease and stroke. The committee also projected an increase in total cardiovascular disease by 3.7% [2]. A separate report from AHA projected health care costs related to cardiovascular risk factors will increase from US $400 billion in 2020 to US $1344 billion in 2050 while accounting for inflation [3]. The AHA supports managing BP as one of 8 strategies for cardiovascular disease prevention [4]. The 2024 Guideline for the Primary Prevention of Stroke recommends screening for hypertension in all adults to identify those at increased risk for stroke [5].

Improved BP control depends on the health care team’s ability to collect, interpret, and act on these findings collectively rather than making the data available to a single clinician. The clinical practice guidelines for cardiovascular disease prevention, management of coronary artery disease, primary and secondary prevention of stroke, management of stroke, and prevention and management of hypertension all explicitly recommend a health care team or team-based care approach [5-10]. In addition, the American College of Cardiology (ACC) Foundation has endorsed ambulatory (clinic-based) BP monitoring by various outpatient health care professionals to improve screening [11]. Pharmacists can play an important role in detecting medication-related causes of suboptimal BP control [12]. Compared with well-resourced patients, underresourced patients may have additional socioeconomic barriers to attending clinic visits, such as transportation, wait times, unknown reasons for visits, adverse clinic experiences, health status, and competing responsibilities [13,14]. Use of remote BP monitoring (RBPM), or monitoring patients using telecommunications (internet, telephone, etc) while patients are outside the health system, may be one method for improving collaboration among health care team members while minimizing the clinical burden on patients. Evidence supporting the use of RPBM for BP control is available, but there are still unresolved challenges with this approach [15].

BP varies in response to genetics and social stressors as well as environmental factors, such as monitoring location and environmental stress [16-18]. Studies that have shown benefits of clinical BP monitoring often do not describe in detail how the BP data were collected, received, cleaned, reported, and acted upon [19-22]. In addition, patients may have difficulty using home BP monitors. The monitors’ properties and features aren’t always chosen based on patient population or the individual patient’s ability to operate the device but instead are chosen based on cost or familiarity. Integration of BP data into the electronic health record (EHR) faces challenges, such as workflow, data compatibility, data autonomy, and EHR security [15].

Dashboards have been developed to monitor BP in patients with dementia [23], renal transplant [24], hypertension [25-29], myocardial infarction [30], heart failure [31-35], diabetes mellitus [36-38], and COVID-19 [39,40], and in patients exposed to pollution [41]. While these examples of other dashboards monitor, track, and trend BP readings, none have been explicitly developed or described to address patient-, provider-, and system-specific challenges. There are multiple RBPM systems available on the market; however, opportunities for end-users to customize these according to patient and provider needs are limited. In addition, many of these systems are embedded in the institution’s EHR rather than as a free-standing web-based application, which makes collaboration across disciplines and institutions difficult. Furthermore, detailed descriptions of the process by which the dashboards were developed and modified are not provided by most of the applications, thereby limiting opportunities for easy adaptation in new systems. The description and design of a dashboard programmed to monitor BP using a BP monitor specifically chosen for stroke survivors has not yet been described in the literature.

Modern technology can address integration challenges related to patient-, provider-, and health system-specific factors in BP management. Real-time remote BP monitoring systems should be selected with consideration of potential barriers. These solutions are accessible to health systems, researchers, and organizations with the financial resources to contract IT solution companies; however, in-house systems can also be developed to meet specific needs.

Patient-specific challenges to remote BP monitoring relate to self-efficacy, motivation, health literacy, digital literacy, functional limitations, wireless access, social support, and data protection [15-18]. Provider-specific factors involve ease of access, data summarization (date and time), data visualization, and identification of outlier values [15,19-22]. System-specific considerations include data aggregation, EHR integration, privacy considerations, data quality and maintenance, system updates, and usage tracking [15].

This paper aims to describe the development and design of a web-based dashboard for clinicians to monitor and manage BP while addressing patient-specific barriers, process transparency, provider challenges, and system constraints. This framework can serve as a blueprint for health systems, researchers, and clinicians seeking to develop in-house remote BP monitoring systems tailored to patient-, provider-, and system-specific needs.

## Methods

### Overview

The dashboard described in this text was developed for use in a clinical trial that incorporates remote BP monitoring and telehealth into the care of stroke survivors (NCT05264298). Briefly, the Video-based Intervention to Address Treatment and Outcome Disparities in Adults Living with transient ischemic attack (TIA) or Stroke (VIRTUAL) trial is a randomized comparative effectiveness study of a multidisciplinary telehealth intervention aimed at improving BP control following stroke. Participants are enrolled during their stroke or TIA hospitalization and randomized to the VIRTUAL intervention or standard care. Participants in the intervention arm receive an RBPM prior to hospital discharge and receive follow-up via telehealth with a stroke provider, pharmacist, and social worker. BP is monitored by a pharmacist who adjusts medications according to a standard protocol. Participants in the standard care arm receive a standard BP monitor with remote monitoring capabilities and receive follow-up care with a stroke provider. Study pharmacists relay patient-reported BP to the treating provider for patients in the standard arm but do not treat elevated BP directly, and thus the dashboard’s primary use is for the intervention group. The intervention lasts for 6 months, and the primary outcome is 6-month 24-hour ambulatory BP.

The study team took the following recommended steps to customize the remote BP monitoring system to meet patient, provider, and system needs

### Patient-Specific Needs

Many patients who have experienced stroke bear lasting physical and cognitive impairment [42,43]. For the VIRTUAL study, the selection of at-home BP monitors surveyed potential options and capabilities to address the needs of stroke survivors who often have physical and cognitive impairment after stroke [44]. Investigators considered application programming interface (API) push ability, transmission modality, ergonomics, reusability, cost, and customer service. The final BP monitor was selected based on these criteria. Patient use of the selected BP device was assessed by the RBPM usage rate (percentage) and the mean number of BP measurements per patient per day over the 180-day intervention period of the VIRTUAL study.

### System-Specific Needs

The principal investigator (AS) contracted with Carematix, the digital health software solutions company (SSC) that distributes the selected monitor for back-end data acquisition. This device is connected to a cellular network and addresses barriers to remote monitoring associated with limited broadband access [44]. Using a cellular network avoids a potentially confusing and complicated internet setup and allows patients without wireless internet access to use the device [44]. Participant BP and heart rate data measured by these devices is transmitted via cellular connection to the SSC’s servers. Using the SSC’s API, the study’s Data Coordinating Center (DCC) developed a web application to accept participant data from the SSC’s servers via an internet connection. The SSC regularly transmits or “pushes” participant data to the DCC’s servers via the API, where it is then stored in the DCC’s database for use in a dashboard displaying patients’ longitudinal BP and heart rate readings. Participant values typically appear in the DCC database and dashboard 2‐5 minutes after readings are taken using the device.

**Figure 1.**
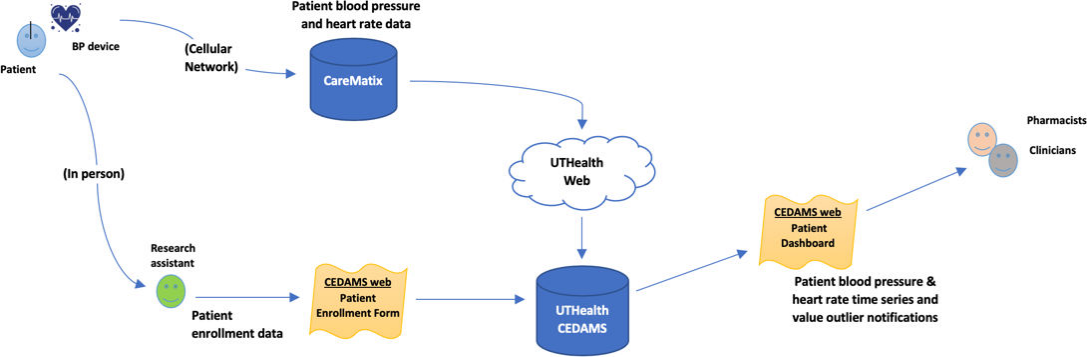
Data map for VIRTUAL Electronic Data Capture System. BP: blood pressure; CEDAMS: Clinical Electronic Data Access and Management System.

The DCC modified an existing, locally developed clinical trial management system (CTMS) called the Clinical Electronic Data Access and Management System (CEDAMS) in close collaboration with clinicians to make the VIRTUAL system. Figure 1 describes the data flow. CEDAMS includes CTMS functionality, electronic Case Report Forms (eCRF), and a dashboard.

In CEDAMS, the participant ID and the device serial number are used to link the data from the device to the patient’s profile in the database. The CTMS manages user accounts and study site information, as well as control over user access permissions, eCRF configuration, and usage monitoring. CEDAMS retrieves patient data from the institution’s database and displays each patient’s BP readings as a time series along with other variables on the dashboard screen. The implementation of CEDAMS was customized for the VIRTUAL trial. System-specific needs were addressed in the design. Alert frequencies, time from alert to notification, and time from data collection to dashboard entry (BP data transmission times) were analyzed as measures of system efficiency.

### Provider-Specific Needs

The development and testing phases followed the DCC’s established protocols for system development. This includes the DCC’s standard operating procedures regarding source code control, nightly system builds, and testing. Separate web servers and databases were used for system development, testing, and production. Testing was performed first by the development staff, then the data coordinating staff, and then the users. The clinician users provided casual feedback regarding the API during this phase, and the feedback was incorporated as appropriate.

The CEDAMS electronic data capture (EDC) system was developed over approximately 3 months, including front-end development, front-end testing, and API development. The data gathered before the API’s completion were then integrated into the completed EDC system at the end of May 2022. Informal user training was provided at clinical research meetings, and a detailed user manual was issued at the launch of the project for clinical users. Provider usage was measured using the number of monthly logins per clinician.

### Ethical Considerations

The VIRTUAL trial was approved by the University of Texas Health Science Center at Houston Committee for the Protection of Human Subjects (study HSC-MS-21‐0549) on August 5, 2021. Patients were provided informed consent, and confidentiality was achieved through the use of generated patient identifiers. Patients were provided compensation in cash per the protocol and were loaned the devices necessary to take and upload readings to the investigators.

## Results

### Patient-Specific Needs

Seventeen BP monitors were considered from 6 different manufacturers and were evaluated based on API push ability, transmission modality, ergonomics, reusability, cost, and customer service. These monitors were further screened for barriers to integrating their data into the EHR data, such as (1) compatibility and synchronicity of data with the EHR; (2) API programming challenges; and (3) the burden on patients to execute the data transfer. Of the 17 monitors, 3 were excluded for lack of API-push ability. Five additional monitors were excluded due to poor ergonomic design. Integration barriers removed another 3 monitors from consideration. The remaining 5 monitors were considered for use in the study, and the Blipcare Blip BP800 distributed by Carematix was determined to be the most suitable for VIRTUAL’s needs based on the previously mentioned criteria. Among participants in the intervention arm, there was 97% engagement with a mean number of 1.3 BP measures per day.

### System-Specific Needs

Figure 1 depicts the VIRTUAL data capture system. The system was designed to operate independently of the EHR to address data security concerns while allowing for potential EHR integration in the future. The system was designed to capture delays in data transmission, identify outliers, and track BP monitoring adherence, enabling effective data quality monitoring. Delays in BP data transmission occurred when the device was turned off before the data could be sent, with transmission resuming the next time the device was powered on. Time from data collection (BP measurement) to transmission to the dashboard database (via the BP device online server) was a median of 1.4 ( IQR 1.0-5.8) minutes. Based on input from clinical providers, BP and heart rate outlier alerts were generated each morning, capturing all outlier readings from the previous day. The average time from a unique alert to provider email notification was 17.9 (range 6.4-30.3) hours. Additional details on alert frequency are provided in Table 1.

**Table 1.** Summary statistics of number of alerts in patients in the intervention group who have been followed up for 6 months (N=190).

Alert type	Number of patients, n (%)	Minimum number of alerts	Maximum number of alerts	Mean (SD)	Median (IQR)
Overall	131 (68.9)	0	109	5.9 (14.4)	2 (0-5.8)
Abnormal systolic BP^a^ (<91 or >179)	82 (43.2)	0	45	1.7 (4.6)	0 (0-2)
High systolic BP (>179)	32 (16.8)	0	44	0.8 (3.6)	0 (0-0)
Low systolic BP (<91)	60 (31.6)	0	24	1 (2.9)	0 (0-1)
Abnormal diastolic BP (<41 or >119)	53 (27.9)	0	80	1.4 (6.6)	0 (0-1)
High diastolic BP (>119)	47 (24.7)	0	80	1.4 (6.6)	0 (0-0)
Low diastolic BP (<41)	7 (3.7)	0	3	0 (0.3)	0 (0-0)
Abnormal heart rate (<50)	59 (31.1)	0	106	2.8 (11.4)	0 (0-1)

a BP: blood pressure.

### Provider-Specific Needs

**Figure 2.**
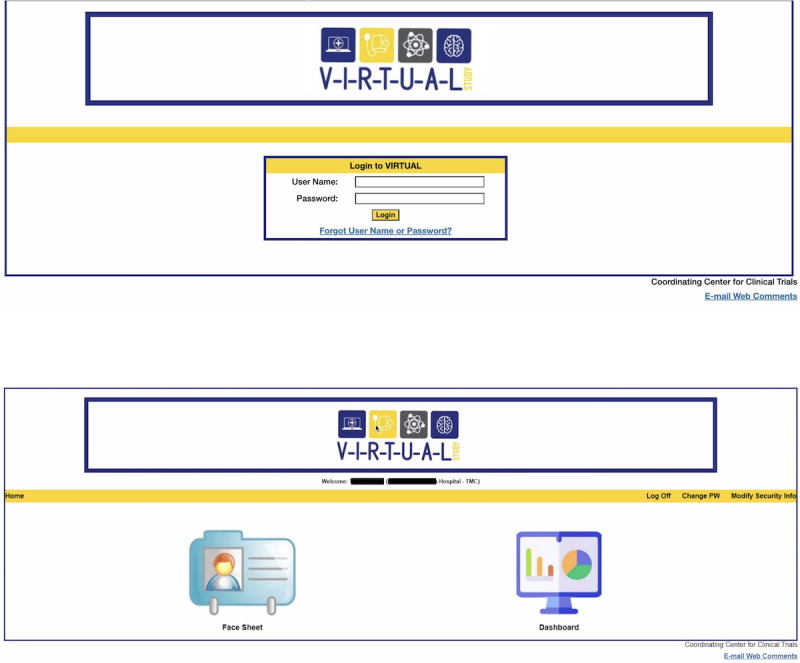
Login page and homepage screens.

The VIRTUAL system was designed with a login page, a home page, a face sheet, and a BP dashboard screen. The login page is the initial page visible to the user with username and password textboxes and is shown in Figure 2. There is a security feature required upon first login to create 3 security questions from a preselected dropdown and a subsequent screen to change the password from the temporary password provided by the DCC. Once logged in, the user is directed to the Home Page which has 2 icons as seen in Multimedia Appendix 1—one for the face sheet and one for the dashboard screen.

Clinical providers provided feedback regarding the need to easily toggle between patient demographic data and BP data and the ability to modify the time window for BP data visualization. The face sheet participant screen displays a list of participants already enrolled and allows the user to add a new participant, select a participant, search participants, and view or sort participants based on participant ID, acrostic participant code, enrollment site, date entered, completion status, assigned clinician, and eCRF reference number Multimedia Appendix 2. The dashboard screen has a dropdown button to select a patient, or the patient’s dashboard screen panel can be accessed from their face sheet. The dashboard screen, as seen in Multimedia Appendix 2, displays systolic BP, diastolic BP, and heart rate during enrollment as well as a 14-day moving average. Each parameter can be selected separately, which removes the other parameters from the chart. The dashboard screen also shows averaged day (08:00-19:59 h), night (<08:00 or >19:59 h), and combined systolic BP, diastolic BP, and heart rates. This component of the dashboard screen can also select day, night, or combined parameters from the chart which will remove the others.

Other CEDAMS VIRTUAL design features include real-time visual cues and email notifications for out-of-range systolic BP, diastolic BP, and heart rate values. Email notifications are sent to appropriate study staff for missing readings when a patient’s device hasn’t sent any data for over 5 days. Also, the system sends reminders to study staff to follow up with patients at specified times after enrollment. The version of the CEDAMS that supports the VIRTUAL trial also supports outbound API function calling to interface with other clinical study support systems such as REDCap® (Research Electronic Data Capture; Vanderbilt University). Having separate systems allowed for the storing of protected health information in CEDAMS and deidentified data in the trial database.

The EDC system successfully performed the intended RBPM functions. The patient-, provider-, and system-specific challenges from traditional monitoring procedures were addressed by the EDC system.

The system was designed to aggregate and summarize data for clinical use. It provides daytime, nighttime, and average readings in both graphical and tabular formats. Pharmacists can adjust the data capture window based on specific needs. Providers receive alerts for out-of-range data, which may prompt changes in medications. Patient and caregiver contact information is securely linked to the capture system to facilitate care. Clinicians effectively used the EDC system to monitor BP. Mean monthly CTMS logins for 4 clinicians were as follows: PharmD1=19, PharmD2=22, PharmD3=29, and PharmD4=44. Alerts for out-of-range BP or heart rate were triggered for 69% (n/N) of patients (Table 1), with most alerts due to abnormal systolic BP (43%), particularly low systolic BP (32%). The distribution of alerts was right-skewed, with an average of 6 alerts per enrolled person and a median of 2 (IQR 2-5.8) alerts per person.

## Discussion

### Principal Findings

Despite the known long-term detrimental effects of uncontrolled BP on cardiovascular and all-cause mortality, more than 50% of US adults, including survivors of stroke, have uncontrolled BP. RBPM has been proven to be effective for BP reduction and is established as a practice for reducing cardiovascular risk and improving team-based BP control in the 2017 ACC/AHA Guideline for the Prevention, Detection, Evaluation, and Management of High Blood Pressure in Adults [10]. However, RBPM has not been routinely implemented into the care of patients, including those at high risk for cardiovascular disease. This is partly due to the lack of published methods for integrating RBPM into clinical practice while addressing patient, provider, and system-level needs and barriers. Herein, we describe steps to developing an RBPM EDC while considering our population’s specific needs.

The monitor was chosen for ease of use in a population of patients with stroke. Among enrolled participants, rates of participation are high, with 97% engagement. The system was also built to address provider needs while overcoming system challenges. The EDC system was successfully developed and implemented within three months to function alongside the EHR without requiring full integration. Clinician feedback shaped features, such as alert timing, customizable visualization windows, and streamlined access to patient data. Clinicians consistently logged in without issues and accessed patients’ readings for monitoring. Patient data were securely protected while remaining accessible to providers. The system supports data monitoring for quality assurance purposes.

### Limitations

This study has several limitations related to its design and measures. We described steps used within a single, large health care system with access to expertise in IT, bioinformatics, health systems research, and clinical trial design. The framework and steps we provide may be more challenging to implement in settings with limited resources. We were limited in some of the data we reported because of the ongoing nature of the clinical trial. The data we can report serve as proxies for system fitness and do not comprehensively address all potential usage barriers faced by patients and providers or integration challenges encountered by the system. Nonetheless, combining these parameters with the context in which they were gathered may offer valuable insights for extrapolation and future exploration.

There were also some data collection limitations of the VIRTUAL EDC system. When taking family members’ BP or when clinic staff demonstrate how to use the BP monitor, the reading is logged as the patient’s BP in the interface. This challenge was mitigated with clinician and patient retraining; however, this can have implications for the duration of clinic visits. We observed delays in transmission from the BP device to the dashboard due to the device being powered off by the user too soon after a reading. While the overall median time for entry into the EDC was minimal, there were some readings that were delayed as a result. For subsequent enrolled patients, we trained them to leave the monitor on for a few minutes after the completion of the reading to complete data transmission.

### Future Directions

Future work in this area could include more human-centered design features to more directly refine the system based on patient, caregiver, and clinician experiences, and test outcomes could include those related to ergonomics and other factors impacting usability. BP monitoring systems can be more specifically tailored to populations and end-users based on different health conditions, environmental factors, and accessibility concerns. Finally, it can be further refined to incorporate clinical decision-making tools, including artificial intelligence to assist with pharmacotherapy recommendations and identify other clinical and social factors related to monitoring adherence and BP control.

### Conclusions

The VIRTUAL system was developed for remote BP monitoring in a population at high risk for cardiovascular disease. Clinicians can analyze, track, and interpret monitored data in near real-time, remotely, which may enhance BP management approaches, particularly in communities with limited access to care. The successful development of this system and its capabilities could serve as a blueprint for creating similar systems for clinical BP monitoring when standard data transparency is insufficient, standard monitor features are incompatible with the user, or when integrating a commercial dashboard may threaten EHR security.

## Supplementary material

10.2196/62700 Multimedia Appendix 1 Face sheet screen and patient information screen.

10.2196/62700 Multimedia Appendix 2 Dashboard screen with data selection function.

## Abbreviations

ACC: American College of Cardiology
AHA: American Heart Association
API: application programming interface
BP: blood pressure
CEDAMS: Clinical Electronic Data Access and Management System
CTMS: clinical trial management system
DCC: data coordinating center
eCRF: electronic case report form
EDC: electronic data capture
EHR: electronic health record
RBPM: remote blood pressure monitoring
REDCap: Research Electronic Data Capture
SSC: software solutions company
TIA: transient ischemic attack
VIRTUAL: Video-based Intervention to Reduce Treatment and Outcome Disparities in Adults Living with Stroke or Transient Ischemic Attack
