# Supplementary figures and images for: Development of a Customizable Web-Based Dashboard for Remote Blood Pressure Monitoring: Feasibility and Usability Implementation Study
Source: JMIR Form Res. 2025 Aug 6;9:e62700. doi: 10.2196/62700 (PMC12327963; doi:10.2196/62700)

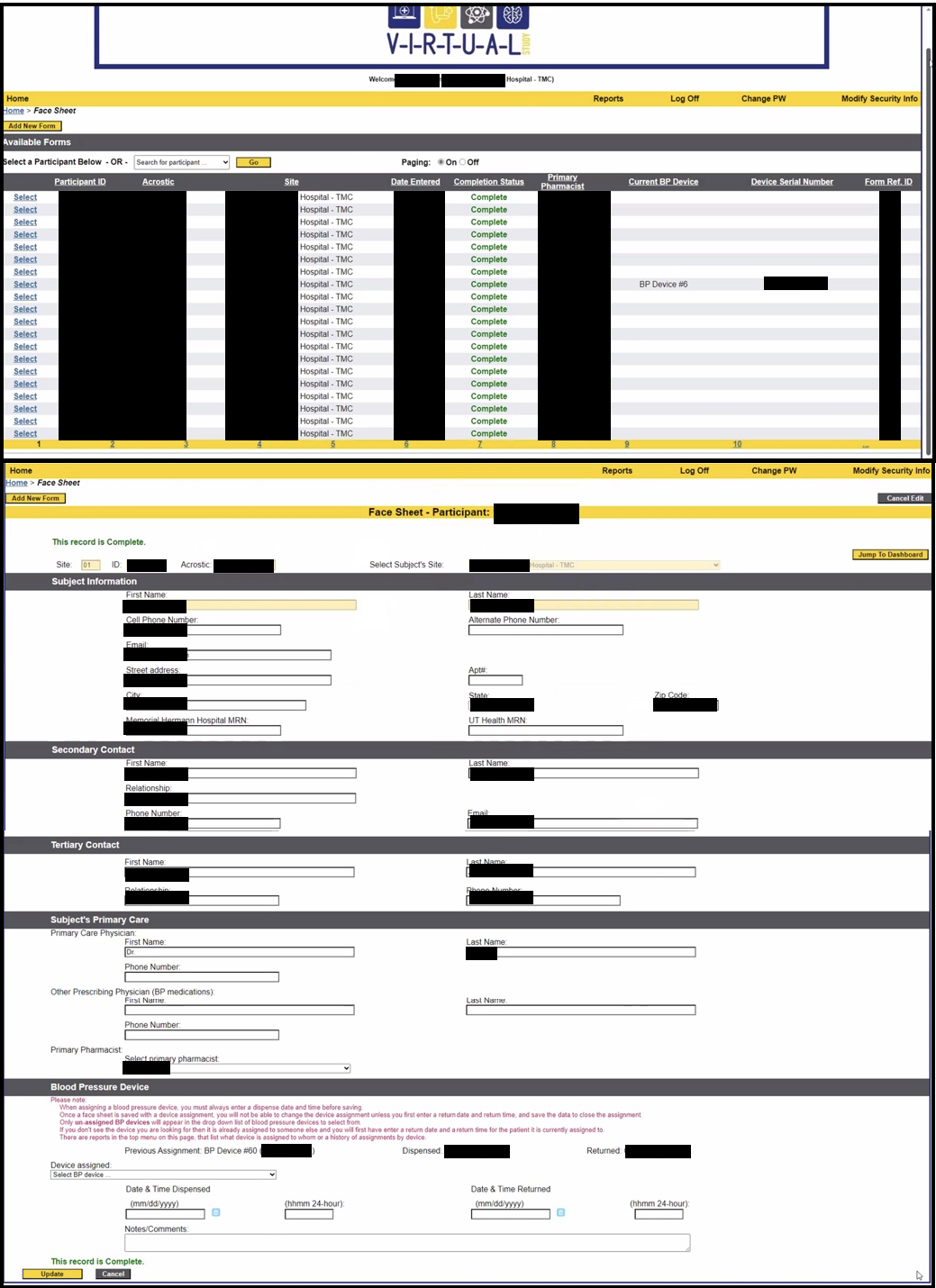

Supplement: Multimedia Appendix 1 [file formative-v9-e62700-s001.png]

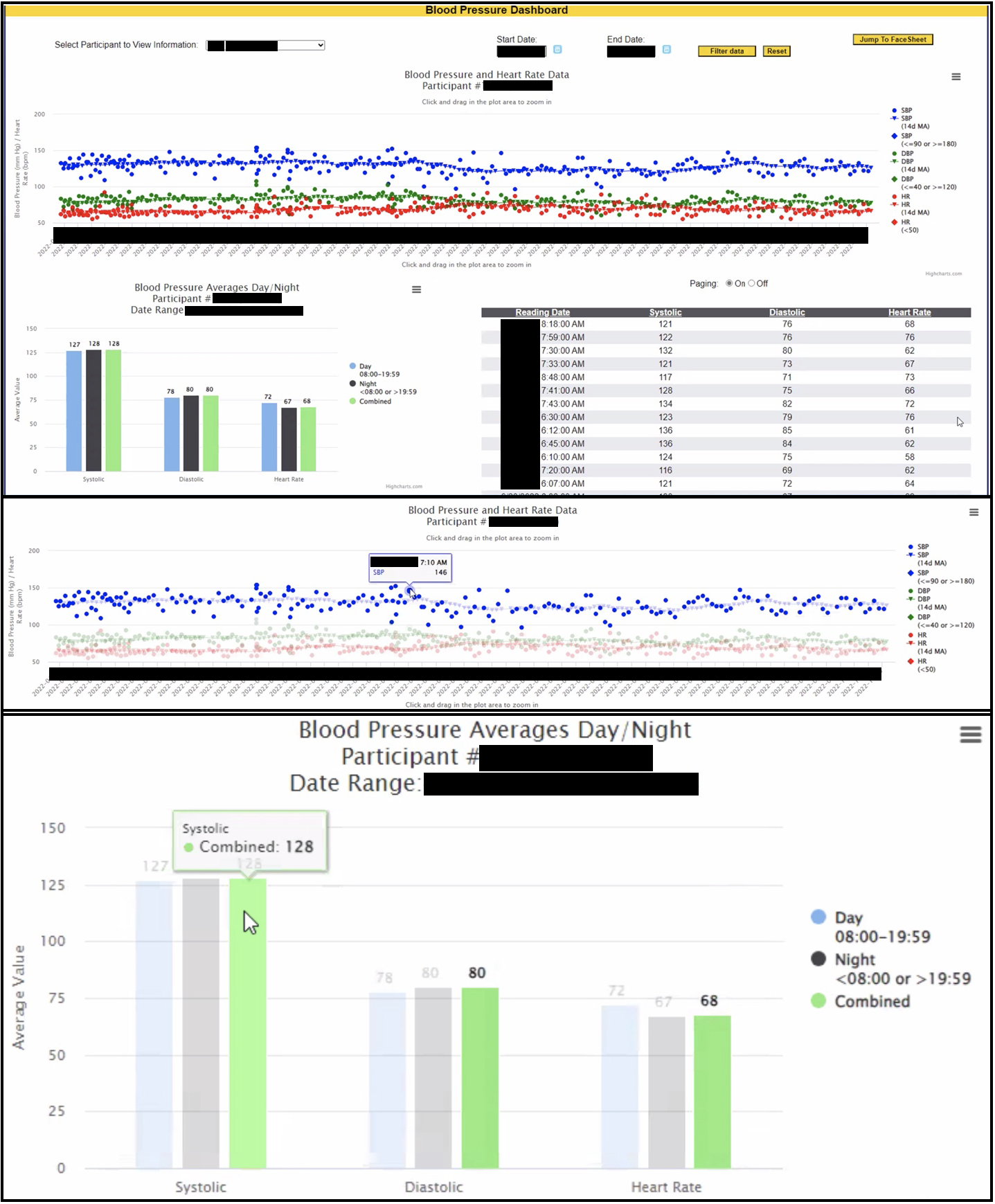

Supplement: Multimedia Appendix 2 [file formative-v9-e62700-s002.png]
